# Rapid Screening of 22 Polycyclic Aromatic Hydrocarbons Residues in Vegetable Oils by Gas Chromatography-Electrostatic Field Orbitrap High Resolution Mass Spectrometry

**DOI:** 10.3389/fnut.2022.949025

**Published:** 2022-06-30

**Authors:** Zhijuan Meng, Sufang Fan, Xiaoxuan Yuan, Qiang Li, Yunxia Huang, Lisha Niu, Guohua Shi, Yan Zhang

**Affiliations:** ^1^Hebei Food Safety Key Laboratory, Key Laboratory of Special Food Supervision Technology for State Market Regulation, Hebei Engineering Research Center for Special Food Safety and Health, Hebei Food Inspection and Research Institute, Shijiazhuang, China; ^2^Hebei Key Laboratory of Forensic Medicine, College of Forensic Medicine, Hebei Medical University, Shijiazhuang, China

**Keywords:** gas chromatography-electrostatic field orbitrap high resolution mass spectrometry (Orbitrap GC-MS), polycyclic aromatic hydrocarbons, vegetable oils, frozen in addition to fat, real sample determination

## Abstract

A method for simultaneous determination of 22 polycyclic aromatic hydrocarbons (PAHs) residues in vegetable oils by gas chromatography-electrostatic field orbitrap high resolution mass spectrometry (Orbitrap GC-MS) was established. The samples were vortexed with acetonitrile, centrifuged at 8,000 r/min for 5 min, and frozen at −70°C for 10 min. The extracts of upper layer were poured out, dried with nitrogen at 40°C, redissolved in dichloromethane, and measured by Orbitrap GC-MS. The matrix interference in vegetable oil could be effectively removed by determining the accurate mass number of target compounds under the full scan mode. Six typical vegetable oil samples (soybean oil, sesame oil, peanut oil, olive oil, rapeseed oil, sunflower oil) were used for method validation. The calibration curve displayed good linearity in the range of 1–100 ng/mL, with correlation coefficients > 0.9950. The limits of detection (LODs) were in the range of 0.10–0.60 μg/kg, and the limits of quantification (LOQs) were in the range of 0.35–2.00 μg/kg. The average spiked recoveries of 22 PAHs in 6 matrices at 5, 50 and 100 μg/kg levels were 76.4–115.4%, and the average relative standard deviations (RSDs) were 1.8–10.8%. The results showed that 22 PAHs were detected in 6 types of 90 edible vegetable oil samples in the Chinese market by this method. Meanwhile, the abundance of light PAHs (LPAHs) was higher than that of heavy PAHs (HPAHs), and its relative contribution of LPAHs to the total PAHs was higher. All levels of BaP conformed to the Chinese requirement of upper limit, 10 μg/kg. However, 13.3 and 11.1% of the samples exceeded the maximum limits of BaP and PAH4 set by EU, 2 and 10 μg/kg, respectively. The total concentrations of 22 PAHs (defined as PAH22) varies greatly among different oil species, and the average PAH22 contents were listed in descending order as follows: peanut oil > sesame oil > olive oil > rapeseed oil > soybean oil > sunflower seed oil. The established method effectively avoided interference from large amounts of lipids and pigments. Therefore, the method is simple, sensitive and suitable for rapid screening and confirmation of PAHs in vegetable oil.

## Introduction

Polycyclic aromatic hydrocarbons (PAHs) are compounds containing more than two benzene rings in the molecule, which include more than 150 compounds such as naphthalene, anthracene, phenanthrene, and pyrene. Benzo[a]pyrene (BaP) was one of the earliest discovered environmental chemical carcinogen, which had strong carcinogenicity hence was often used as the representative of PAHs (1). Meanwhile, some of them were considered to be possible or probable human carcinogens (2–6). Edible oil, as an indispensable cooking material in people's daily life, played a pivotal role in life (7, 8). However, it has been reported that edible oils are more susceptible to PAHs contamination, and the absorption of PAHs contained in oils will increase in the intestinal tract, which can seriously harm human health. PAHs pollution in edible oil mainly came from the migration of pollutants in raw materials, processing and packaging materials (9–11). At present, the upper residue level for BaP was set at 10 mg/kg in edible oils according to the China national standard (12). Meanwhile, the European Food Safety Authority Panel on Contaminants in the Food Chain demonstrated that BaP was not a sufficient surrogate of PAHs existence in food and recommended the sum of four PAHs (PAH4) (BaP, benzo[a]anthracene, chrysene and benzo[b]fluoranthene) as evaluation indicators. Recently, Commission Regulation of European Union No 835/2011 established the maximum legislation levels of BaP and PAH4 for edible oils, which were 2 and 10 μg/kg, respectively (13). And the detection method of PAH in edible oil under China national standard only involved 16 species (14). PAHs can be divided into light polycyclic aromatic hydrocarbons (LPAHs) containing 2–4 aromatic rings, and heavy polycyclic aromatic hydrocarbons (HPAHs) containing five or more aromatic rings. The carcinogenicity of PAHs increased with the number of benzene rings (13–16). Therefore, it is of great significance to study a variety of efficient PAH detection methods to more comprehensively monitor and evaluate the PAHs pollution in edible oils, and ensure the quality and safety of edible oils.

Lipophilic compounds in vegetable oils are easily co-extracted with the target PAHs, which affects the accurate qualitative and quantitative analysis of PAHs. PAHs in vegetable oils usually needed to be extracted with solvent, and then purified by gel-permeation chromatography (17, 18), solid-phase extraction (SPE) (19–21), solid-phase microextraction (22). The methods are relatively cumbersome and expensive, and they are impossible to achieve efficient and rapid detection of PAHs in vegetable oil. Freeze-defatting is a simple and low-cost method which can effectively remove fat and other macromolecular interfering substances in the sample. The detection of PAHs mainly included high performance liquid chromatography (HPLC) (23, 24), gas chromatography-mass spectrometry (GC-MS) (9, 25), liquid chromatography-tandem mass spectrometry (LC-MS/MS) (26) and gas chromatography-tandem mass spectrometry (GC-MS/MS) (27). HPLC and GC-MS are more commonly used, and they are also adopted in China national standard. The practical application of capillary electrophoresis is relatively rare. LC-MS/MS and GC-MS/MS provide new and effective methods for the detection of PAH due to their better selectivity and detection ability. The use of ordinary mass spectrometry has limitations on account of the complex matrix and high interference of vegetable oil. And it is prone to produce false positives and misjudgments. High resolution mass spectrometry, such as gas chromatography-electrostatic field orbitrap high resolution mass spectrometry (Orbitrap GC-MS) has the characteristics of high resolution and high sensitivity. Moreover, it is not limited by the number of compounds. It also could collect more comprehensive compound information, and could quickly and accurately screen and quantify target compounds at low levels in complex matrices (28). Its high resolution enables better separation of target compounds from interfering impurities. What is more, its high sensitivity ensures effective detection of low-concentration compounds. At present, Orbitrap GC-MS technology had been used in pharmaceutical research (29), environmental analysis (30) and pesticide residues in fruits and vegetables (31). However, the use of Orbitrap GC-MS to determine PAHs in vegetable oils is rarely reported.

In this study, a new method was developed for the determination of 22 PAHs in edible vegetable oils by Orbitrap GC-MS after removing major lipophilic interfering compounds through freezing at ultra-low temperature. It was proven that this method could be used as an accurate and quantitative technique for routine analysis. The collection mode allows non-target analysis using databases and/or libraries, as well as retrospective analysis. The data collection has no relationship with the number of compounds in the database so that the data can be reviewed and reanalyzed to expand the target range. At the same time, satisfactory results were also obtained in the verification of actual samples. The method has the characteristics of fast detection speed, high throughput, accurate and reliable detection results, and provides a scientific basis and a new method for national supervision.

## Materials and Methods

### Materials and Reagents

Soybean oil, peanut oil, olive oil, rapeseed oil, sesame oil, sunflower seed oil and vegetable oil samples were purchased from China local supermarkets and sealed at room temperature for use later.

Acetonitrile and dichloromethane (both chromatographically pure) was purchased from Merck (Darmstadt, Germany). The standard substances of twenty-two PAEs (mixed standard solutions with mass concentration of 1,000 μg/mL, and dichloromethane as the solvent) were purchased from Alta Scientific Co., Ltd. Stock solutions (Tianjin, China): Naphthalene (Nap), Acenaphthylene (Acy), Acenaphthene (Ace), Fluorene (Fl), Phenanthrene (Phe), Anthracene (Ant), Fluoranthene (Flu), Pyrene (Pyr), Benz[a]anthracene (BaA), Chrysene (Chr), 5-Methylchrysene (5-Mchr), Benzo[b]fluoranthene (BbFlu), Benzo[k]fluoranthene (BkFlu), Benzo[e]pyrene (BeP), Benzo[a]pyrene (BaP), Benzo[g,h,i]perylene (BghiP), Indeno[1,2,3-cd]pyrene (IP), Dibenz[a,h]anthracene (DBahA), Dibenzo[a,l]pyrene (DBalP), Dibenzo[a,e]pyrene (DBaeP), Dibenzo[a,i]pyrene (DBaiP), Dibenzo(a,h)pyrene (DBahP).

### Instruments and Equipment

Orbitrap GC-MS (Thermo Fisher Scientific, USA), 3K15 high-speed refrigerated centrifuge (Sigma, Germany), Vortex mixer (IKA, Germany), Refrigerator (Qingdao Haier Co., Ltd., China) were used in our experiment.

### Orbitrap GC-MS Analytical Conditions

A Orbitrap GC-MS consisting of an AI/AS 1310 autosampler, a TRACE 1300 Series GC with a hot split/splitless injector, an electron impact ion source (EI), and a hybrid quadrupole Orbitrap mass spectrometer with an HCD (higher energy collision-induced dissociation) cell, was used. The column was set at a constant flow rate of 1.0 mL/min using helium as carrier gas (purity ≥ 99.999%). GC separation was performed on a 30 m × 0.25 mm ID, 0.25 μm HP-5MS column (5% phenyl-95% methylpolysiloxane, Agilent Scientific, USA). The column temperature program started from 80°C (hold 2 min), increased to 260°C at the rate of 8°C /min (hold 5 min), then increased to 310°C at the rate of 8°C /min, and held at this final temperature for 10 min. The temperature of the injector port was 280°C, and an aliquot of 1 μL of the sample was injected in the splitless mode. The flow rate of helium carrier gas was 1.0 mL/min. EI was performed at 70 eV, with the ion source and transfer line temperature at 280°C.

All data with m/z range of 50–500 were acquired in full scan mode. The nitrogen gas supply for the C-trap and HCD cell was 5.0 grade (99.999%, Linde gas). In the method, the Orbitrap resolving power was set at 60,000 FWHM (200 m/z) and the automatic gain control (AGC) target was set at 3e6. The actual scan speed under these conditions was ~4 scans/s. External mass calibration was performed before each sequence using perfluorotributylamine (68.9945, 99.9928, 130.9911, 196.9827, 218.9846, 263.9860, 413.9760, and 501.9694) with a mass error tolerance of ±1 ppm (±0.2 mDa), and internal mass calibration was carried out in the instrument using three background ions from the column bleed as lock mass (C_5_H_15_O_3_${\text{Si}}_{3}^{+}$, 207.03236; C_7_H_21_O_4_${\text{Si}}_{4}^{+}$, 281.05115; C_9_H_27_O_5_${\text{Si}}_{5}^{+}$, 355.06994) with a search window of ±2 ppm (±1 mDa) during the measurement. If none of the three specified background ions were found within their exact mass ±2 ppm in a certain scan, no internal locking was applied for that scan. The instrument was controlled using Tune 2.8 and Trace Finder 4.1 (Thermo Scientific).

#### Preparation of Standard Solutions

One milliliter of the mixed standard solution was pipetted. Each mixed standard solution was placed into a 100-mL volumetric flask and diluted with dichloromethane, and then stored as a stock solution in a brown storage bottle at −20°C. It could be used after being placed at room temperature for 30 min. The stock solution was diluted with dichloromethane to prepare intermediate working solutions with mass concentrations of 1 and 0.1 μg/mL, respectively, which were used for addition and recovery experiments and preparation of standard working solutions. The mixed standard intermediate working solution was diluted with dichloromethane to obtain mass concentrations of 1, 5, 10, 20, 50, and 100 ng/mL, respectively. Then they were stored in the refrigerator at 4°C until use.

#### Sample Pretreatment

A total of 0.5 g of vegetable oil sample (±0.001 g) were weighted accurately and placed into a 15 mL centrifuge tube, then 3 mL of acetonitrile were accurately added, vortex was carried out for 2 min, and then this solution centrifuge at 8,000 r/min for 3 min. The extraction processes were repeated twice, and the supernatants from the two extractions were combined and placed in a −70°C ultra-low temperature freezer for 10 min. The supernatant was purifed under nitrogen blowing to nearly dry in a water bath at 40°C, and 1 mL of dichloromethane was added, vortexed for 5 s, transferred to a sample vial, and sent to Orbitrap GC-MS for analysis. For the reagent blank, the operations were the same except that no sample was added.

#### Database Creation

In this experiment, 22 PAHs compounds were selected and prepared into 1.0 μg/mL mixed standard solutions. The retention time of the corresponding compound, the accurate molecular weight, and the chemical formula of the fragment ions were obtained under the full scan mode. Three fragment ions of each compound were selected to obtain ion information (accurate mass and chemical formula). The data were inputted into Trace Finder (4.1) software to establish the relevant database. The Trace Finder software could not only realize the rapid batch and automatic processing of data, but also set the functions of qualitative, quantitative and method establishment. According to the established database, it could realize the rapid screening of target substances. The database mainly contains the compounds name, CAS registration number, fragment ion information, retention time, rings and other information (Table 1).

**Table 1:** Formula, Retention time and Mass spectrometric of the 22 PAHs.

**PAH**	**CAS** ** number**	**Chemical** ** formula**	**Molecular** ** weigh**	**Rings**	**Retention** ** time/min**	**Quantitative** ** ion (*m/z*)**	**Qualitative** ** ion (*m/z*)**	**Qualitative** ** ion (*m/z*)**
Nap	91-20-3	C10H8	128	2	7.46	128.06205	129.06541	102.04640
Acy	208-96-8	C12H8	152	3	11.93	152.06205	153.06541	126.04640
Ace	83-32-9	C12H10	154	3	12.48	154.07770	152.06205	153.06988
Fl	86-73-7	C13H10	166	3	13.97	166.07770	165.06988	139.05423
Phe	85-01-8	C14H10	178	3	16.75	178.07770	152.06205	179.08106
Ant	120-12-7	C14H10	178	3	16.89	178.07770	176.06205	152.06205
Flu	206-44-0	C16H10	202	4	20.28	202.07770	200.06205	101.03858
Pyr	129-00-0	C16H10	202	4	20.90	202.07770	200.06205	203.08106
BaA	56-55-3	C18H12	228	4	24.51	228.09325	226.07770	209.09669
Chr	218-01-9	C18H12	228	4	24.62	228.09335	226.07770	113.03858
5-Mchr	3697-24-3	C19H14	242	4	26.14	242.10900	241.10118	226.07770
BbFlu	205-99-2	C20H12	252	5	28.54	252.09335	250.07770	125.03858
BkFlu	207-08-9	C20H12	252	5	28.57	252.09335	250.07770	126.04640
BeP	192-97-2	C20H12	252	5	29.79	252.09335	250.07770	113.03858
BaP	50-32-8	C20H12	252	5	30.03	252.09335	250.07770	125.03864
BghiP	191-24-2	C22H12	276	6	34.34	276.09335	274.07770	138.04640
IP	193-39-5	C22H12	276	6	34.49	278.10895	276.09335	274.07770
DBahA	53-70-3	C22H14	278	5	35.05	276.09335	274.07770	138.04640
DBalP	191-30-0	C24H14	302	6	38.57	302.10886	300.09332	150.04643
DBaeP	192-65-4	C24H14	302	6	39.82	302.10886	300.09332	225.04292
DBaiP	189-55-9	C24H14	302	6	40.35	302.10886	300.09332	281.05121
DBahP	189-64-0	C24H14	302	6	40.37	302.10886	300.09332	253.01678

#### Validation of the Analytical Procedure

A validation study was performed in terms of matrix effects, linearity, LODs, LODs, recovery, as well as intra-day and inter-day precision. The recoveries and precision of the method were determined by analyzing the average of six replicates of spiked blank matrix at concentration levels of 5, 50, and 100 μg/kg. The multi-standard working solution with concentration of 0.18–100 ng/mL were used to evaluate the linearity of the method. Spiked samples were used to test the LODs and LOQs, the levels of spiked analytes with signal-to-noise ratio of 3 and 10 were defined as the LOD and LOQ of the method. To determine the matrix effects, the slopes of the calibration curves obtained in matrix matched standards were compared with those acquired from the solvent-based standards. The intra-day and inter-day precision of the method were tested using the soybean oil and sesame oil samples at vial concentrations of 5 and 50 μg/kg, which were used as the repeatability and reproducibility of the method, respectively. More specifically, the repeatability analysis was performed by preparing and analyzing six identical samples in 1 day, and the reproducibility analysis was performed by preparing and analyzing one sample on six different days.

## Results and Discussion

### Selection of Extraction Conditions

PAHs are insoluble in water, but soluble in most organic solvents. According to the method in Section Sample Pretreatment. Dichloromethane, n-hexane, acetonitrile and n-hexane + acetonitrile were used for extraction, and the effects of different extraction solvents on the recovery rate of 22 PAHs (addition amount of 10 μg/kg) were compared. The results showed that there were many co-extraction substances in dichloromethane, which had an impact on the result. The matrix effect was serious, and the recovery rate was low. Meanwhile, fat has high solubility in n-hexane, and its interference effect is serious. Acetonitrile could precipitate proteins and has a good degreasing effect. Moreover, some lipophilic compounds, such as triglycerides, diglycerides, fatty acids and other substances could still be extracted by acetonitrile from the vegetable oil although the solubility of fat in acetonitrile is low, which might bring trouble to the subsequent purification. In this study, an ultra-low temperature freezer at −70°C was used for freezing. It was found that organic extraction solvent and oil were both liquid when the freezing time was <5 min and the organic extraction solvent and oil were solid when the freezing time was longer than 15 min. The oil of lower layer remained solid and the extract of upper layer was still liquid when the freezing time was about 10 min. Therefore, the freezing time was determined to be 10 min. After freezing, the extract of upper layer can be easily separated and poured into a centrifuge tube to achieve full separation from oil. The remaining part after the nitrogen blowing is also an important factor affecting the detection of PAH. The losses of Nap and BaA are more serious, while the Acy, Ace and Flu also have certain losses when the temperature is too high. The above losses can be effectively avoided at temperature of 40°C. Therefore, the oil samples were extract by acetonitrile, then the supernatant was frozen at −70°C for 10 min. After the solidified oil of lower layer was removed, the supernatant was taken to be purified, which could effectively avoid the interference of oil on the sample. In this way, the spiked recoveries of the 22 PAHs in the samples were all higher than 75%.

### Comparison of Sample Pretreatment Between This Method and the China National Standard Method

At present, the detection of polycyclic aromatic hydrocarbons in food is mainly based on the China national standard GB 5009.265-2021 (14), which includes two types, HPLC fluorescence and GC-MS method. The former one has high sensitivity but not suitable for the detection of acenaphthene because of its weak fluorescence absorption. Moreover, the target peak is easily interfered by impurity peaks, and the vegetable oil sample matrix is more complex, making it difficult to guarantee the accuracy of qualitative and quantitative analysis. In contrast, the latter one is generally more complicated and has lower recovery rate. The procedure of GC-MS method includes saponification with potassium hydroxide ethanol solution, purified by SPE column, diluting to volume with acetone-iso-octane after nitrogen blowing, and sample injection on the machine. During the experimental process, there is need to activate the SPE purification column with a lot of reagents, and this operation is complicated and time-consuming. The determination method in this experiment combined freeze-defatting purification and Orbitrap GC-MS, specifically using acetonitrile vortex extraction, freeze defatting purification, nitrogen blowing for concentration, and on-machine measurement. This method shortens the time of pretreatment, and utilizes the qualitative and quantitative accuracy of Orbitrap GC-MS to achieve the purpose of high-throughput detection. Its ability to resist matrix interference is higher than that of single quadrupole, and it can accurately qualitatively and quantitatively analyze PAHs, which is suitable for rapid screening and analysis of PAHs in vegetable oil.

### Optimization of Mass Spectrometry Conditions

**Figure 1:**
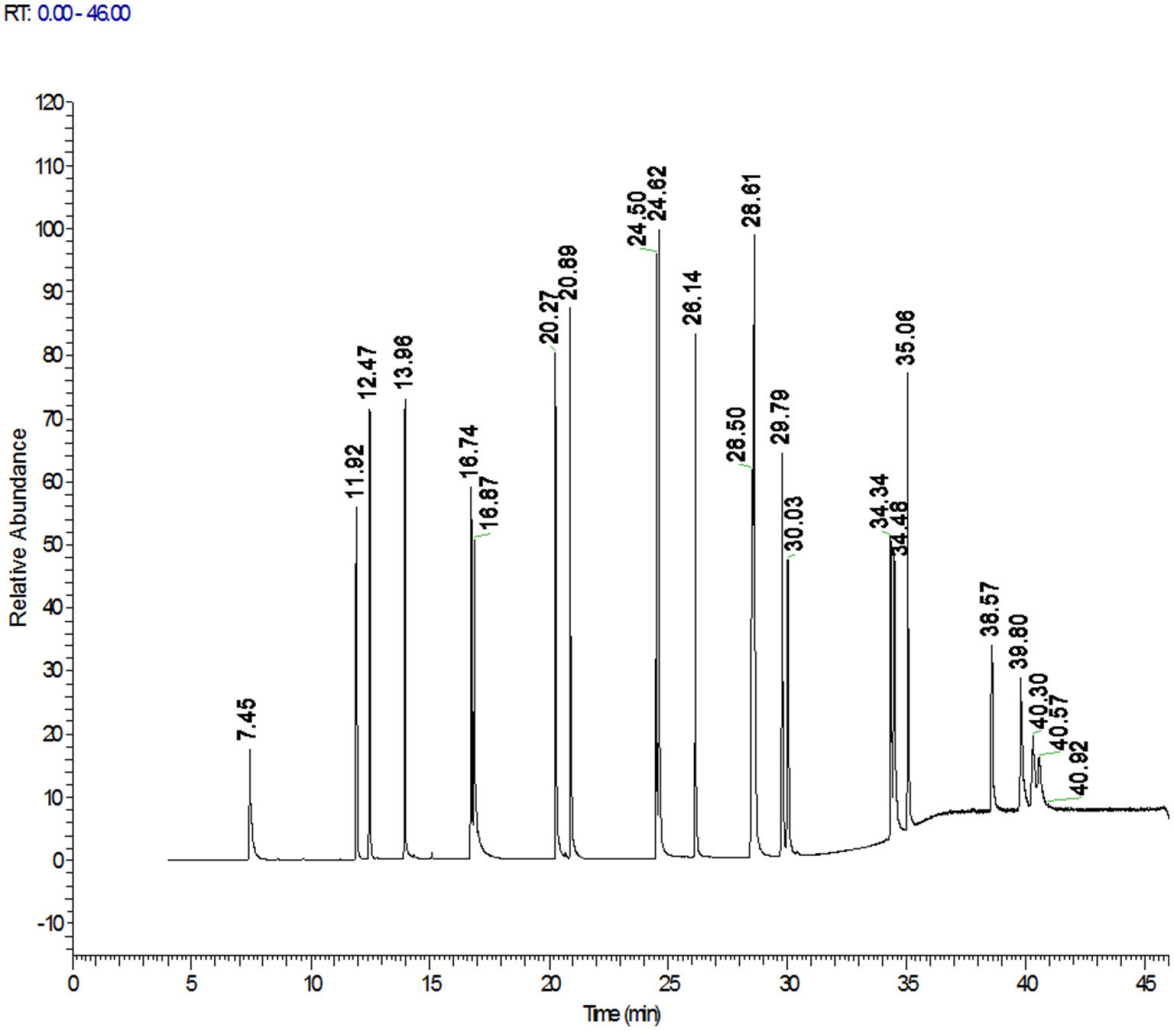
Total ion chromatograms of the 22 PAHs.

The high-resolution mass spectrometer is different from the triple quadrupole quantification method. The Orbitrap GC-MS used in this study adopts the full scan mode, which simplifies the optimization of gas chromatography-mass spectrometry parameters, saves the sample preparation time of the pretreatment, makes the screening and accurate quantitative analysis more convenient, and improves the work efficiency and accuracy. Accurate mass of the target compound was obtained by full scan. The exact mass of 3 fragment ions of 22 PAHs are listed in Table 1, which can be kept to 5 decimal places and can be effectively and accurately distinguished from interfering substances. One was used as the quantification ion, and the other two were used as the qualitative ions. This study found that matrix interference was significantly reduced and the screening accuracy was also improved when the resolution of R ≥ 60,000 was used in sample detection. All the analyte targets could be clearly distinguished from the interferences in the matrix, and PAHs could be quickly determined and accurately quantified to improve the reliability of test results. The total ion chromatograms of 22 PAHs were shown in Figure 1.

### Qualitative Screening Confirmation and Retrospective Analysis

The main factors affecting the qualitative determination included the exact mass deviation, retention time deviation, isotope distribution and isotope abundance ratio of the compounds when searching with the spectral library established in Table 1. According to EU SANTE/11945/2015 (32), at least 1 accurate mass ion and 1 fragment ion were required for confirmation using high-resolution mass spectrometry.

**Figure 2:**
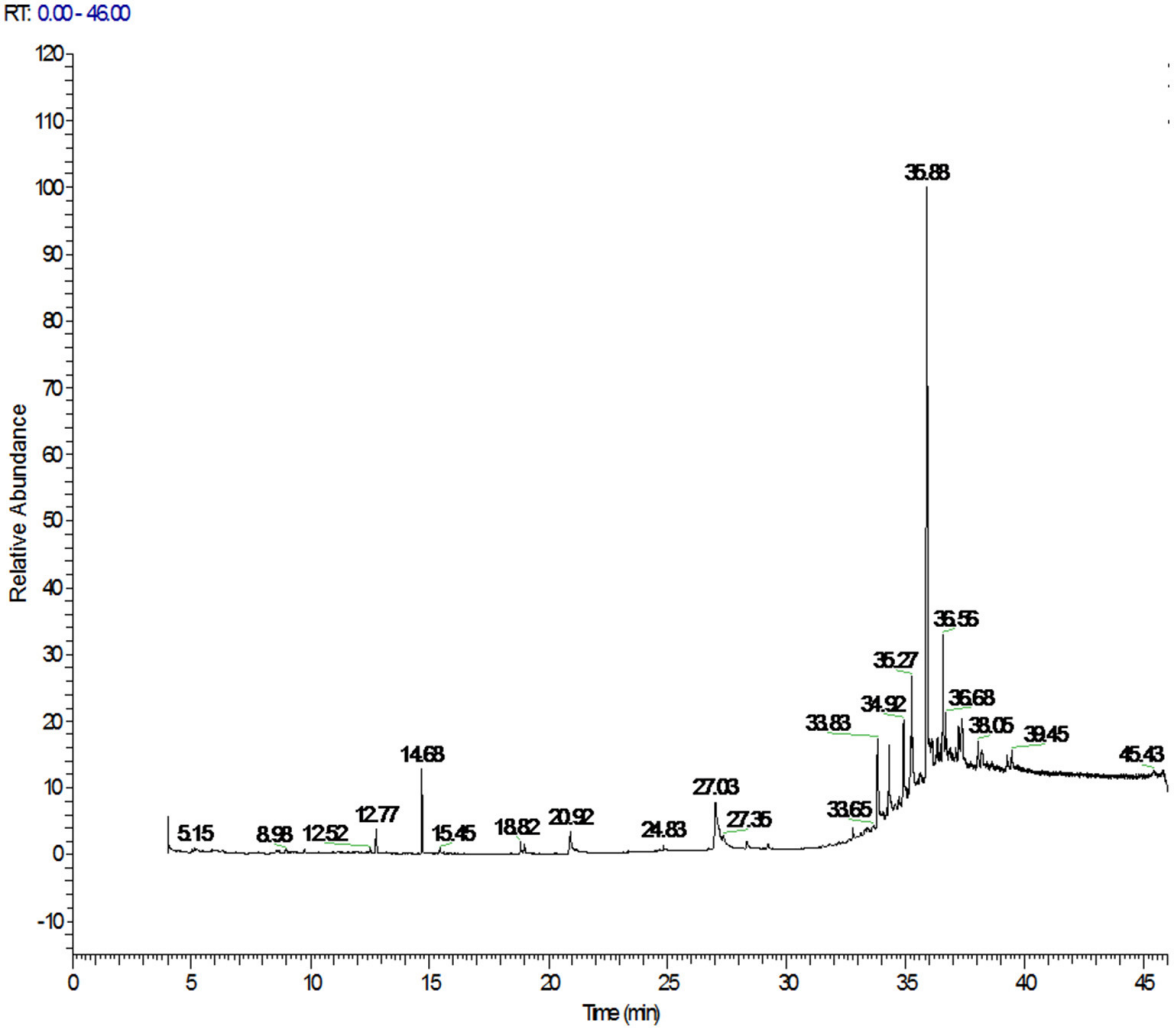
Total ion chromatograms of PAHs in positive soybean oil sample.

**Figure 3:**
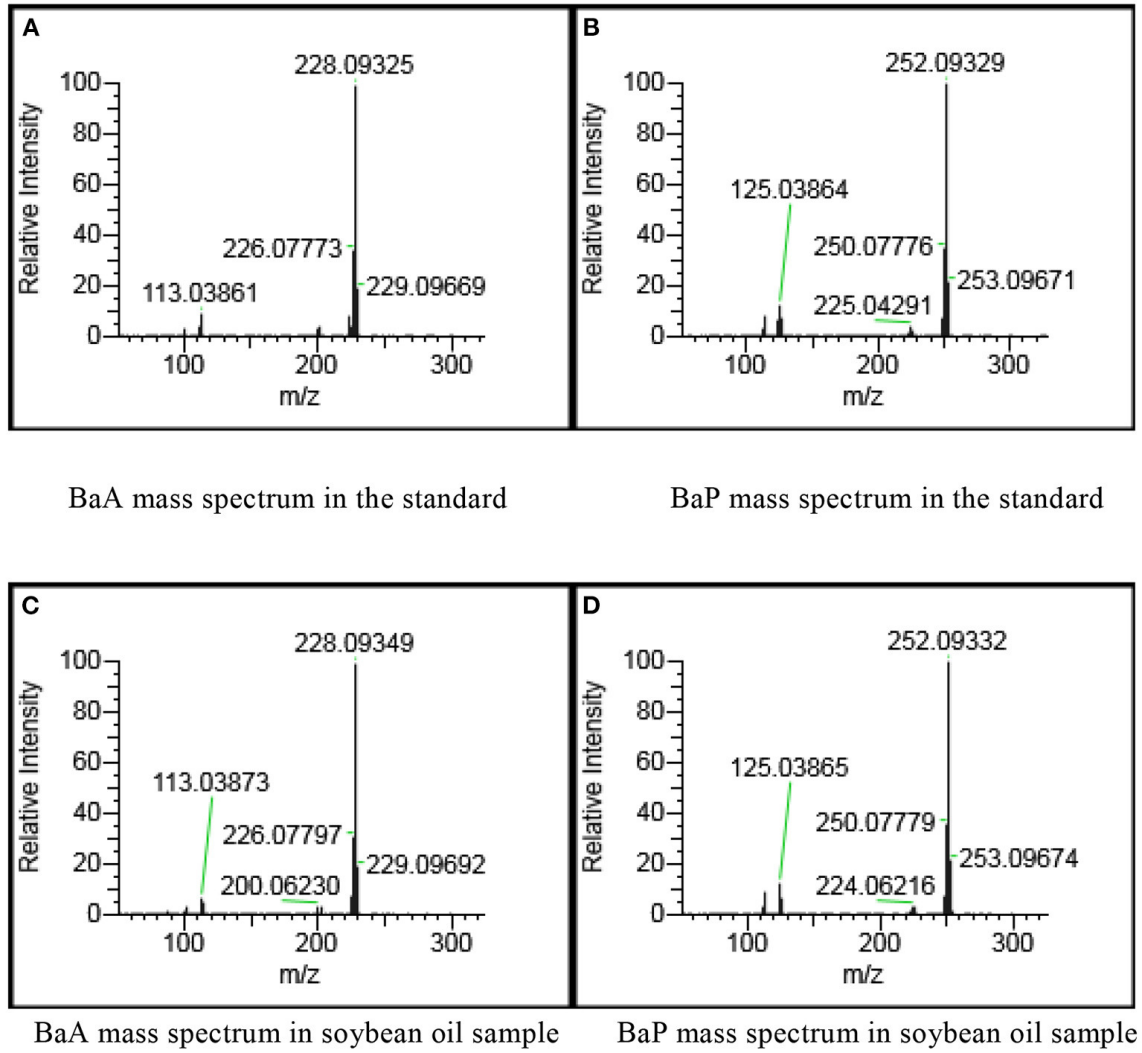
Mass spectrogram of reference materials and positive soybean oil sample at 50 μg/kg. **(A)** BaA mass spectrum in the standard. **(B)** BaP mass spectrum in the standard. **(C)** BaA mass spectrum in soybean oil sample. **(D)** BaP mass spectrum in soybean oil sample.

The PAHs target compounds could be accurately identified using the spectral library established in this paper. The total ion chromatograms of PAHs in positive soybean oil sample were shown in Figure 2 which contained rich chemical information. However, the chromatographic peak of the target could not be seen. This information was inputted into the qualitative and quantitative analysis software for analysis so as to achieve the purpose of confirming the target and accurate quantification. Figure 3 showed the mass spectrogram of reference materials and positive soybean oil sample. The qualitative and quantitative ions in the positive sample were consistent with those in the standard, and the presence of the compound could be determined. This demonstrates that Orbitrap GC-MS can extract useful information from the experiment of complex matrices, highlighting its advantages in high-throughput screening.

Orbitrap GC-MS often collects the full spectrum and can collect data more comprehensively. The data collection is independent of the number of compounds in the database so that the data can be reviewed and re-analyzed after collection to expand the target range. For the analysis of samples of new compounds, such as Cyclopenta[cd]pyrene (CPcdP), the retention time, molecular formula, exact molecular mass and CAS number of CPcdP were added to the 22 PAHs database. This method can expand and analyze target compounds without re-collecting real-time data. It has flexibility, which is convenient for high-throughput screening and quantitative analysis of PAHs. This method could be adopted in vegetable oil risk monitoring technology.

### Methodological Validation

#### Matrix Effect

As we all know, the co-elution of matrix constituents can interfere with the ionization of the target compounds, causing ion enhancement or suppression. The presence of matrix effects was perceived as a signal enhancement or suppression of the analytes, which plays an important role in the quality of the quantitative data obtained from the method. In the present study, the matrix effect was considered to be ignored if the slope ratios of matrix/solvent were in the range of 0.9–1.1, while it would be regarded as matrix suppression effect if the value was lower than 0.9, and it would be regarded as a matrix enhancement effect when the value was larger than 1.1. The experimental results showed that the effects of 22 PAHs in 6 kinds of vegetable oil samples were different, and the range of variation was 0.91–1.08. The target to be tested has a certain matrix effect in six kinds of matrix, however the effect is not obvious. Therefore, in this method dichloromethane is used to prepare a series of standard working solutions for quantitative analysis.

#### Linear Range, LODs, LOQs, and Intra-Day and Inter-day Precision for the Method

Orbitrap GC-MS was used for qualitative and quantitative analysis of 22 kinds of PAHs. The curve was plotted with peak area (y-axis) against the concentration (x-axis). The results of mass spectrometry demonstrated that the R-squared of 22 PAHs were no < 0.9950. The LODs and LOQs of the method were determined by the addition of blank samples. The LODs and LOQs for the 22 PAHs were found to be 0.10–0.60 μg/kg and 0.35–2.0 μg/kg. It is superior to the national standard method (22) and can meet the testing requirements. The related parameters are listed in Table 2. Information of Table 3 in which precision values were expressed as intra-day (*n* = 6) and inter-day (6 days) precision for the chromatographic method was included in the data. The RSDs of repeatability and reproducibility were found to be within in the ranges 1.7–6.4% and 2.3–9.2% in both matrices, respectively.

**Table 2:** Linear equations, correlation coefficients, linear ranges, LODs and LOQs of the 22 PAHs.

**PAH**	**Linear equation**	**R^**2**^**	**Linear range/** **(ng/mL)**	**LOD/(μg/kg)**	**LOQ/(μg/kg)**
Nap	*Y* = 3.482 × 10^8^*X* −4.320 × 10^6^	0.9998	1.40–100	0.40	1.40
Acy	*Y* = 4.037 × 10^8^*X* −5.528 × 10^6^	0.9993	0.35–100	0.10	0.35
Ace	*Y* = 3.259 × 10^8^*X* −2.456 × 10^6^	0.9996	0.35–100	0.10	0.35
Fl	*Y* = 4.612 × 10^8^*X* −2.094 × 10^7^	0.9991	0.35–100	0.10	0.35
Phe	*Y* = 6.313 × 10^8^*X* −6.452 × 10^6^	0.9995	0.70–100	0.20	0.70
Ant	*Y* = 3.953 × 10^8^*X* −8.159 × 10^5^	0.9977	0.70–100	0.20	0.70
Flu	*Y* = 4.538 × 10^8^*X* −1.437 × 10^7^	0.9995	0.35–100	0.10	0.35
Pyr	*Y* = 7.560 × 10^7^*X* −1.651 × 10^6^	0.9980	0.35–100	0.10	0.35
BaA	*Y* = 5.501 × 10^8^*X* −1.299 × 10^7^	0.9995	0.35–100	0.10	0.35
Chr	*Y* = 5.360 × 10^8^*X* −3.304 × 10^7^	0.9993	0.35–100	0.10	0.35
5-Mer	*Y* = 6.422 × 10^8^*X* −1.383 × 10^7^	0.9973	0.35–100	0.10	0.35
BbFlu	*Y* = 3.057 × 10^8^*X* −2.642 × 10^6^	0.9982	0.35–100	0.10	0.35
BkFlu	*Y* = 5.564 × 10^8^*X* −1.284 × 10^7^	0.9983	0.35–100	0.10	0.35
BeP	*Y* = 4.652 × 10^8^*X* −5.661 × 10^6^	0.9991	0.70–100	0.20	0.70
BaP	*Y* = 3.472 × 10^8^*X* −1.561 × 10^7^	0.9961	0.70–100	0.20	0.70
BghiP	*Y* = 3.064 × 10^8^*X* −2.157 × 10^5^	0.9992	1.00–100	0.30	1.00
IP	*Y* = 6.064 × 10^8^*X* −1.553 × 10^7^	0.9984	1.00–100	0.30	1.00
DBahA	*Y* = 5.064 × 10^8^*X* −1.258 × 10^7^	0.9991	1.00–100	0.30	1.00
DBalP	*Y* = 4.064 × 10^8^*X* −6.069 × 10^6^	0.9990	1.60–100	0.50	1.60
DBaeP	*Y* = 3.074 × 10^8^*X* −5.235 × 10^7^	0.9951	1.60–100	0.50	1.60
DBaiP	*Y* = 5.054 × 10^8^*X* −3.456 × 10^7^	0.9952	2.00–100	0.60	2.00
DBahP	*Y* = 3.729 × 10^8^*X* −4.176 × 10^7^	0.9961	2.00–100	0.60	2.00

**Table 3:** Precision of the method in soybean oil and sesame oil at levels of 5 and 50 μg/kg.

**PAE**	**Intra-day variability (RSDs/%)**				**Inter-day variability (RSDs/%)**			
	**5** **μg/kg**		**50** **μg/kg**		**5** **μg/kg**		**50** **μg/kg**	
	**Soybean oil**	**Sesame oil**	**Soybean oil**	**Sesame oil**	**Soybean oil**	**Sesame oil**	**Soybean oil**	**Sesame oil**
Nap	3.2	3.6	1.7	5.8	8.2	5.6	7.6	6.5
Acy	1.7	1.9	1.9	1.9	6.4	8.7	4.6	9.1
Ace	5.5	6.4	2.7	3.7	7.6	6.5	9.0	4.5
Fl	2.6	2.9	2.5	2.9	5.2	6.4	4.5	8.3
Phe	1.9	3.5	2.8	2.6	5.5	8.1	6.6	4.5
Ant	3.5	1.8	2.6	1.8	4.9	8.4	7.4	4.6
Flu	1.8	1.7	1.9	2.6	3.7	5.4	9.6	5.3
Pyr	1.9	2.8	3.7	2.8	9.2	6.7	6.6	2.8
BaA	3.5	2.9	2.8	2.5	6.5	6.4	8.5	6.5
Chr	2.8	2.4	2.5	2.8	7.5	3.5	5.4	4.6
5-Mer	4.4	4.7	1.8	1.9	6.4	7.5	6.5	7.6
BbFlu	1.8	1.8	1.9	1.8	9.0	6.4	5.6	6.4
BkFlu	1.9	2.4	1.7	3.8	6.4	6.6	8.4	8.1
BeP	1.7	1.8	4.8	3.5	5.3	5.8	7.5	4.8
BaP	6.7	5.6	3.9	6.3	8.3	5.5	3.3	5.7
BghiP	4.5	1.9	2.7	6.0	2.3	9.1	8.2	6.4
IP	2.7	2.7	2.5	3.5	10	3.4	7.3	5.5
DBahA	1.8	3.9	2.8	1.9	4.4	7.4	6.4	4.5
DBalP	3.4	1.8	3.4	4.4	7.5	8.5	7.5	5.7
DBaeP	2.6	5.6	2.9	5.7	8.3	4.5	4.6	6.7
DBaiP	3.7	3.7	1.8	4.8	5.4	3.5	3.4	3.4
DBahP	4.8	3.5	3.6	1.8	6.8	3.5	4.3	5.4

#### Recovery and Precision

In this experiment, soybean oil, sesame oil, sunflower seed oil, peanut oil, olive oil, and rapeseed oil were selected for standard addition and determination so as to investigate the precision and recovery rate of the method. The sensitivity of the proposed method was significantly improved because the matrix interference was reduced. Recovery and repeatability experiments were performed at three levels (5, 50, and 100 μg/kg) with six replicates at each level to evaluate the accuracy and precision of the methods. The accuracy was estimated by recoveries (%) and the precision was evaluated by RSDs (%) of the spiked samples. The results were shown in Table 4, which showed that the mean recoveries of 22 PAHs were 85.1–115.4% except that the recoveries of DBaiP and DBahP ranged from 79.2 to 82.2% and 76.4 to 80.4% respectively at three levels. Additionally, the average RSDs were 1.8–10.8%. It shows that the method has good recovery and repeatability, and can meet the requirements of daily monitoring of 22 PAHs in vegetable oil samples, and the data are accurate and reliable.

**Table 4:** Recoveries and RSDs of soybean oil, sesame oil, sunflower seed oil, peanut oil, olive oil, and rapeseed oil samples at three spiked levels of the 22 PAHs.

**PAH**	**Spiked/** **(μg/kg)**	**Soybean oil** **Recoveries/** **% (RSDs/%)**	**Sesame oil** **Recoveries/** **% (RSDs/%)**	**Sunflower seed oil** **Recoveries/** **% (RSDs/%)**	**Peanut oil** **Recoveries/** **% (RSDs/%)**	**Olive oil** **Recoveries/** **% (RSDs/%)**	**Rapeseed oil** **Recoveries/** **% (RSDs/%)**
Nap	5	88.5 (9.8)	88.5 (10.8)	102.4 (9.6)	87.5 (7.2)	86.5 (6.8)	108.5 (5.6)
	50	85.3 (9.1)	94.2 (4.5)	96.5 (7.4)	85.3 (8.2)	85.9 (5.1)	86.3 (7.2)
	100	86.6 (7.1)	95.6 (9.7)	98.4 (7.2)	85.6 (5.5)	86.4 (5.6)	98.6 (3.4)
Acy	5	90.2 (8.2)	87.4 (8.2)	88.4 (9.1)	89.2 (8.6)	88.2 (7.4)	85.2 (5.7)
	50	89.4 (6.1)	92.7 (4.8)	94.2 (9.5)	85.4 (5.9)	86.4 (3.8)	86.4 (8.2)
	100	91.8 (7.2)	85.1 (9.7)	92.8 (9.2)	85.1 (6.5)	88.8 (2.8)	97.8 (6.6)
Ace	5	95.2 (6.1)	94.4 (6.3)	90.4 (9.5)	91.2 (2.3)	92.2 (6.4)	85.2 (4.3)
	50	92.2 (7.2)	88.5 (8.1)	101.3 (4.2)	93.2 (3.4)	91.2 (5.2)	89.2 (2.8)
	100	93.3 (9.2)	97.1 (4.4)	92.3 (8.7)	88.3 (4.4)	90.3 (6.2)	91.3 (4.3)
Fl	5	97.1 (8.2)	93.5 (5.3)	93.5 (8.9)	92.1 (2.9)	97.1 (8.7)	87.1 (2.2)
	50	94.2 (7.1)	102.4 (9.4)	97.2 (7.7)	93.2 (3.6)	95.2 (7.6)	90.2 (6.5)
	100	91.2 (6.2)	96.5 (7.1)	108.4 (6.5)	89.2 (5.4)	93.2 (6.2)	91.2 (4.9)
Phe	5	101.1 (7.1)	98.4 (7.2)	103.4 (8.2)	102.1 (6.1)	99.1 (8.1)	108.1 (8.5)
	50	107.3 (1.2)	103.4 (9.1)	89.3 (2.4)	104.3 (7.4)	97.3 (4.2)	105.3 (7.6)
	100	103.1 (7.1)	97.2 (9.4)	88.3 (9.5)	102.1 (5.2)	102.1 (5.1)	104.1 (5.9)
Ant	5	86.1 (7.1)	102.8 (9.2)	93.5 (5.2)	87.1 (5.9)	85.1 (6.1)	87.1 (4.6)
	50	86.2 (5.2)	90.4 (9.5)	105.2 (4.4)	85.2 (4.7)	89.2 (4.2)	85.2 (5.6)
	100	85.9 (5.1)	80.3 (4.3)	95.6 (4.5)	88.5 (1.9)	88.9 (5.8)	86.9 (7.7)
Flu	5	89 (9.1)	92.3 (8.5)	91.2 (9.5)	91.2 (7.7)	91.2 (5.1)	91.2 (4.3)
	50	93.2 (8.1)	93.5 (8.6)	90.4 (4.2)	88.2 (5.9)	93.2 (6.1)	96.2 (5.2)
	100	89.3 (8.2)	97.2 (7.4)	94.2 (4.5)	88.9 (4.5)	89.3 (8.9)	88.3 (6.6)
Pyr	5	92.2 (6.1)	108.4 (6.3)	105.6 (9.7)	92.2 (6.3)	93.2 (4.1)	90.2 (7.2)
	50	92.8 (7.2)	93.4 (8.9)	92.1 (4.2)	94.8 (2.9)	94.8 (7.9)	91.8 (4.5)
	100	95.2 (6.1)	89.3 (2.2)	104.1 (4.0)	93.2 (7.2)	96.2 (6.5)	93.2 (3.8)
BaA	5	102.2 (7.2)	89.3 (9.3)	95.3 (6.5)	104.2 (4.3)	99.2 (7.4)	104.2 (4.9)
	50	103.3 (7.2)	93.5 (5.4)	88.3 (4.4)	102.3 (6.6)	100.3 (5.2)	115.4 (6.7)
	100	104.1 (8.2)	105.2 (4.4)	95.4 (6.2)	103.1 (7.8)	102.1 (6.8)	102.1 (5.6)
Chr	5	85.9 (7.1)	95.6 (4.4)	92.2 (7.1)	87.4 (5.9)	94.9 (5.9)	86.9 (4.4)
	50	86.2 (6.2)	87.5 (7.4)	92.3 (7.3)	86.5 (6.9)	89.2 (6.4)	88.2 (5.9)
	100	89.1 (7.1)	87.1 (6.2)	99.3 (8.3)	90.1 (5.9)	87.1 (7.5)	89.1 (5.6)
5-Mer	5	107.3 (8.2)	106.1 (6.4)	107.6 (11.2)	104.3 (4.6)	105.3 (5.2)	97.3 (4.8)
	50	104.1 (7.1)	89.4 (5.2)	109.5 (9.8)	102.5 (3.6)	102.4 (4.1)	104.1 (8.1)
	100	108.1 (7.1)	87.5 (7.2)	95.5 (9.0)	105.1 (5.4)	103.1 (6.1)	98.1 (4.8)
BbFlu	5	86.2 (5.2)	108.3 (9.2)	86.7 (6.8)	86.2 (2.9)	86.2 (3.2)	86.2 (5.6)
	50	85.2 (7.1)	86.8 (9.1)	96.5 (8.3)	97.2 (4.3)	85.2 (7.8)	89.2 (7.2)
	100	88.1 (9.8)	112.6 (8.2)	82.8 (7.4)	86.1 (3.8)	87.1 (5.5)	85.1 (5.6)
BkFlu	5	89.2 (5.3)	87.4 (7.3)	98.1 (8.2)	88.2 (4.3)	86.2 (6.1)	89.5 (7.2)
	50	93.2 (5.1)	109.1 (9.1)	89.2 (5.5)	93.2 (6.1)	98.2 (5.2)	96.2 (5.9)
	100	95.1 (4.0)	93.3 (9.1)	93.2 (6.6)	94.1 (5.3)	94.1 (4.5)	92.1 (4.3)
BeP	5	99.2 (7.3)	98.0 (6.2)	95.3 (4.2)	97.2 (2.3)	94.2 (3.3)	99.1 (5.6)
	50	105.2 (7.3)	104.2 (9.3)	98.5 (7.7)	104.2 (6.3)	98.2 (9.8)	104.2 (4.8)
	100	103.2 (8.2)	98.3 (8.2)	88.5 (7.5)	107.2 (6.2)	103.2 (6.2)	102.2 (5.5)
BaP	5	90.3 (7.2)	89.1 (9.3)	106.5 (7.4)	106.3 (7.8)	91.5 (8.2)	100.6 (4.8)
	50	101.2 (9.3)	102.4 (9.4)	94.5 (5.3)	92.2 (9.4)	102.2 (6.3)	92.2 (7.4)
	100	100.2 (8.3)	96.5 (7.1)	96.9 (1.8)	101.2 (8.2)	93.4 (4.3)	103.1 (5.9)
BghiP	5	91.2 (8.3)	98.4 (7.2)	104.2 (8.3)	101.2 (5.3)	94.2 (7.3)	103.2 (6.8)
	50	86.2 (5.3)	88.4 (9.1)	95.5 (4.0)	86.5 (4.3)	87.2 (6.3)	86.2 (5.8)
	100	88.1 (2.2)	97.2 (9.4)	97.2 (6.3)	89.1 (6.2)	91.1 (4.1)	89.1 (4.6)
IP	5	91.3 (5.3)	92.8 (9.2)	90.3 (8.1)	90.3 (4.3)	92.3 (5.8)	91.5 (5.7)
	50	89.4 (8.1)	90.4 (9.5)	107.5 (9.2)	89.4 (5.1)	89.4 (6.4)	92.4 (5.9)
	100	92.1 (8.2)	100.3 (4.3)	92.3 (7.5)	90.1 (3.2)	93.1 (7.2)	97.1 (4.9)
DBahA	5	87.3 (7.4)	102.3 (8.5)	103.5 (9.3)	94.5 (9.2)	92.5 (8.4)	101.3 (4.4)
	50	89.2 (9.1)	93.5 (8.6)	110.3 (8.5)	102.6 (8.1)	103.3 (8.5)	88.2 (4.5)
	100	93.4 (9.2)	97.2 (7.4)	90.2 (9.1)	104.2 (7.3)	96.3 (7.1)	94.2 (8.2)
DBalP	5	90.5 (9.6)	98.4 (6.3)	95.2 (6.5)	115.1 (6.4)	113.5 (9.1)	100.2 (7.4)
	50	85.5 (9.3)	103.4 (8.9)	92.5 (5.5)	92.9 (9.2)	113.1 (8.2)	99.3 (6.4)
	100	93.2 (9.1)	91.2 (9.4)	95.5 (8.7)	102.3 (4.2)	121.3 (9.2)	92.1 (9.1)
DBaeP	5	94.3 (9.4)	90.4 (4.5)	87.4 (4.3)	87.8 (4.4)	97.9 (6.3)	88.6 (8.2)
	50	95.6 (9.7)	94.2 (4.6)	95.1 (6.4)	95.4 (6.7)	108.5 (9.6)	102.4 (8.2)
	100	107.2 (9.3)	105.6 (9.3)	108.8 (9.2)	89.6 (7.5)	111.4 (6.3)	100.5 (5.2)
DBaiP	5	80.2 (9.4)	80.4 (8.2)	79.2 (9.8)	80.4 (6.2)	80.3 (4.6)	80.4 (9.8)
	50	79.5 (9.6)	82.1 (4.1)	82.2 (5.4)	79.4 (7.8)	79.1 (6.3)	79.3 (9.1)
	100	78.8 (9.2)	81.6 (9.7)	79.2 (8.2)	81.6 (6.7)	81.5 (8.2)	78.5 (6.4)
DBahP	5	79.5 (9.1)	76.4 (6.4)	79.5 (6.3)	76.5 (7.4)	78.5 (8.2)	76.4 (7.5)
	50	80.2 (9.5)	78.5 (8.1)	77.5 (7.4)	79.3 (3.1)	78.4 (5.2)	78.3 (6.2)
	100	80.4 (4.2)	77.1 (4.8)	76.4 (6.3)	80.1 (9.6)	76.4 (7.2)	79.1 (4.2)

### Determination of Actual Samples

A total of 90 samples of 6 edible vegetable oils collected from the Chinese market were analyzed in this study, including rapeseed oil (*n* = 10), soybean oil (*n* = 12), olive oil (*n* = 12), peanut oil (*n* = 20), sunflower seed oil (*n* = 18) and sesame oil (*n* = 18). The method established in this experiment can determine low levels of PAHs, and has the advantages of accurate qualitative and quantitative characteristics. It can fully meets the needs of actual sample screening.

The detection range of individual PAHs and the concentration range of LPAHs, HPAHs, PAH4, and PAH22 were determined by the type of edible oil studied. Twenty-two analytes were detected in all oil samples. The content of PAH22 in 90 oil samples ranged from 7.38 to 143.25 μg/kg, with an average concentration of 42.03 μg/kg. The BaP concentration ranged from 0.15 to 8.43 μg/kg, with an average concentration of 1.52 μg/kg. The variation range of PAH4 content was 1.24–25.34 μg/kg, and the average concentration was 2.03 μg/kg. The results showed that Phe, Fluo, and Pyr were the three compounds that has the highest concentrations. DbaA had the relatively lowest concentrations, with an average concentration of 0.35 μg/kg. The concentrations of BaP of tested oils (0.15–8.43 μg/kg) were all conformed to the upper limit, 10 mg/kg, set by China. However, in the 90 edible oils tested, a total of 12 oil samples (2 canola oil, 4 peanut oil and 6 sesame oil) exceeded the EU Commission's maximum limit of Bap, 2 μg/kg, and 10 samples (7 peanut oil and 2 sesame oil) exceeded the EU Commission's limit of PAH4, 10 μg/kg.

In order to assess the individual contributions of the 22 PAHs more reasonably, the entire PAHs group was divided into two parts, LPAHs and HPAHs. The total concentration of LPAHs and HPAHs were calculated. LPAHs were the major contaminants in all tested oil samples, and as mentioned above, Phe, Fluo, and Pyr were the major contaminants in edible oils. Since the abundance of LPAHs in vegetable oil samples was higher than that of HPAHs, the relative contribution of LPAHs to total PAHs was higher. The concentration of LPAHs in the 90 oil samples ranged from 5.42 to 132.34 μg/kg, with an average concentration of 38.78 μg/kg. The concentration of HPAHs ranged from 0.85 to 27.03 μg/kg, with an average concentration of 6.58 μg/kg. Since ~90% of the PAH content comes from the contribution of LPAHs, the distribution of total PAHs observed in our study is similar to that of LPAHs, while the contribution of HPAHs is much smaller (about 10%). However, most carcinogens (BaP and DBahA) were included in HPAHs and their relative carcinogenicity is extremely high. In consequence, the presence of HPAHs in edible oil still needs special attention. Therefore, measuring LPAHs or HPAHs alone cannot fully evaluate the characteristics of PAHs content in edible vegetable oils, and an overall evaluation of both is required.

In this study, PAHs in the 90 vegetable oils tested of different types were different. Peanut oil and sesame oil were the two most polluted oils, and soybean oil and sunflower seed oil were the two least polluted species. The average concentration of PAH22 in the tested oil samples was as follows: peanut oil > sesame oil > olive oil > rapeseed oil > soybean oil > sunflower seed oil. The contents of BaP, PAH4, and PAH22 in peanut oil were 0.34–6.21, 2.45–30.25, and 39.42–162.43 μg/kg, respectively. The contents of BaP, PAH4, and PAH22 in sesame oil were 0.21–2.31, 2.08–8.56, and 11.32–38.08 μg/kg, respectively. Peanut oil and sesame oil showed higher levels of PAHs than other edible vegetable oils. It can be seen that there is great variability in the levels of PAHs among different kinds of vegetable oils. Reasonable explanations for the higher PAHs contamination levels in peanut oil and sesame oil are as follows: in order to maintain the flavor of peanut oil and sesame oil, only a cold filtration process was used in the refining process to remove colloidal impurities such as phospholipids, therefore, the PAHs contamination in peanut oil and sesame was not eliminated during roasting, and these compounds would still remain in the final oil.

## Conclusion

In this experiment, a method for the analysis of 22 kinds of PAHs in vegetable oils was established by the combination of defatting purification with Orbitrap GC-MS. Retrospective analyses (post-target and non-target screenings) was performed by taking advantage of the high sensitivity of the Orbitrap analyzer when operating in full scan mode, and the valuable accurate mass information provided. This method has the advantages of simple pretreatment, high purification efficiency, high throughput, and accurate analysis. The validated data showed the suitability of the applied method for the determination of 22 PAHs in vegetable oils. It provides an important technology for the determination of trace PAHs in vegetable oil samples.

Twenty-two PAHs in 6 types of edible vegetable oils from the Chinese market were determined. The abundance of LPAHs is higher than that of HPAHs, and the relative contribution rate of LPAHs to the total PAHs is higher. The determined levels of BaP met the upper level, 10 mg/kg, set in China national standard. However, 13.3 and 11.1% of the samples exceeded the EU-regulated maximum limits for BaP and PAH4, 2 and 10 μg/kg, respectively. The content of PAH22 varies greatly among different oil species, and the average PAH22 content is listed in descending order as follows: peanut oil > sesame oil > olive oil > rapeseed oil > soybean oil > sunflower seed oil. Findings of this study will help us to better understand the risk characteristics of PAHs in vegetable oil. Moreover, its application will provide regulators a useful guide to update the information in the context of risk evaluation.

## Data Availability Statement

The original contributions presented in the study are included in the article/supplementary material, further inquiries can be directed to the corresponding authors.

## Author Contributions

GS and YZ conceived and designed the experiments. ZM, SF, YH, and LN performed the experiments. QL analyzed the data. XY wrote the original draft. All authors have read and approved the manuscript.

## Funding

This work was supported by National Key Research and Development Program of China (Project No. 2019YFC1606400).

## Conflict of Interest

The authors declare that the research was conducted in the absence of any commercial or financial relationships that could be construed as a potential conflict of interest.

## Publisher's Note

All claims expressed in this article are solely those of the authors and do not necessarily represent those of their affiliated organizations, or those of the publisher, the editors and the reviewers. Any product that may be evaluated in this article, or claim that may be made by its manufacturer, is not guaranteed or endorsed by the publisher.
